# Biomarker reveals HIV's hidden reservoir

**DOI:** 10.7554/eLife.04742

**Published:** 2014-10-16

**Authors:** Leslie R Cockerham, Steven G Deeks

**Affiliations:** 1 **Leslie R Cockerham** is in the HIV/AIDS Division, San Francisco General Hospital, University of California, San Francisco, United States; 2 **Steven G Deeks** is in the HIV/AIDS Division, San Francisco General Hospital, University of California, San Francisco, United StatesSDeeks@php.ucsf.edu

**Keywords:** HIV-1, reservoir, antiretroviral therapy, cure, primary infection, Human

## Abstract

Determining the total amount of HIV DNA in people undergoing antiretroviral therapy could accelerate the development of novel therapies and potential cures for HIV infection.

**Related research article** Williams JP, Hurst J, Stöhr W, Robinson N, Brown H, Fisher M, Kinloch S, Cooper D, Schechter M, Tambussi G, Fidler S, Carrington M, Babiker A, Weber J, Koelsch KK, Kelleher AD, Phillips RE, Frater J, on behalf of the SPARTAC Trial Investigators. 2014. HIV-1 DNA predicts disease progression and post-treatment virological control. *eLife* **3**:e03821. doi: 10.7554/eLife.03821

**Image** HIV (yellow) can hide in, and later re-emerge from, infected white blood cells (blue). Image credit: NIAID

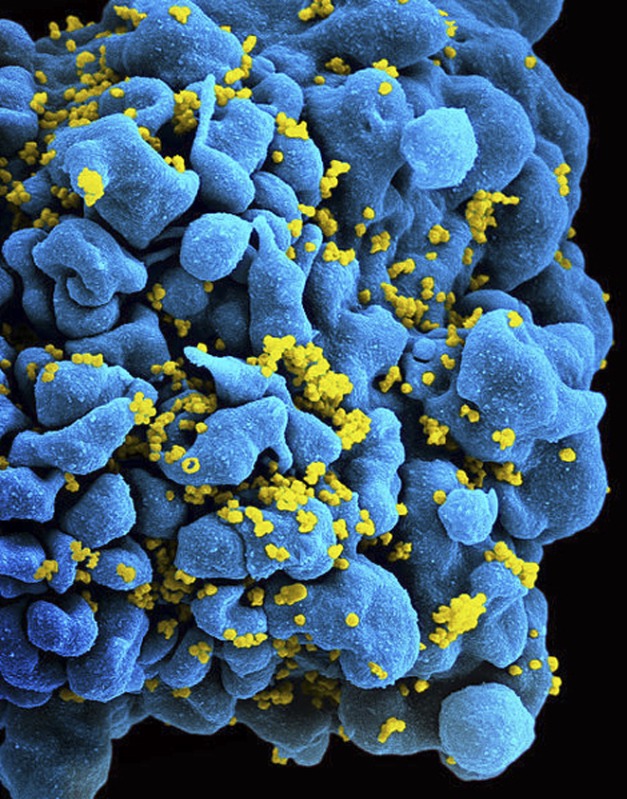

HIV is a virus that hijacks the immune system's white blood cells and forces them to make new copies of the virus. Treatment with antiretroviral drugs is remarkably effective at suppressing replication of the virus, and most individuals who take antiretroviral drugs are able to reduce the amount of HIV circulating in their bloodstream to undetectable levels. However, this treatment cannot eliminate a small but stubborn population of infected white blood cells that contain a copy of the virus's genetic material within their own genomes. These cells can persist in the body for life, and they allow the virus to re-emerge and ignite new rounds of infection if antiretroviral therapy (or ART) is ever interrupted. Eliminating these cells is the focus of an emerging global effort aimed at finding a cure for HIV disease.

In order to eradicate these cells, we first need to be able to measure them accurately. Most people on long-term ART have about 10–1000 copies of HIV DNA per million white blood cells that can be infected by the virus (Eriksson et al., 2013). However, most of this ‘total HIV DNA’ is defective and cannot trigger new infections (See Figure 1A). The rare cells that appear to contain intact copies of the virus, which are able to replicate, are referred to as the ‘latent reservoir’. Moreover, only a fraction of the cells in the latent reservoir can be induced to produce new viruses in cell culture experiments (Ho et al., 2013). Developing a high-throughput method for measuring the size of the latent reservoir will therefore require us to identify a specific molecule (or biomarker) that distinguishes the cells in the reservoir from other infected cells. In the absence of such a method, the only way to currently determine if an intervention has effectively cured a patient, and reduced or eliminated the reservoir, is to interrupt therapy under the supervision of a physician and wait until the virus re-emerges or ‘rebounds’ (Figure 1B). This approach clearly carries some risk to the patient (and his or her sexual partners) and is logistically challenging.

**Figure 1.**
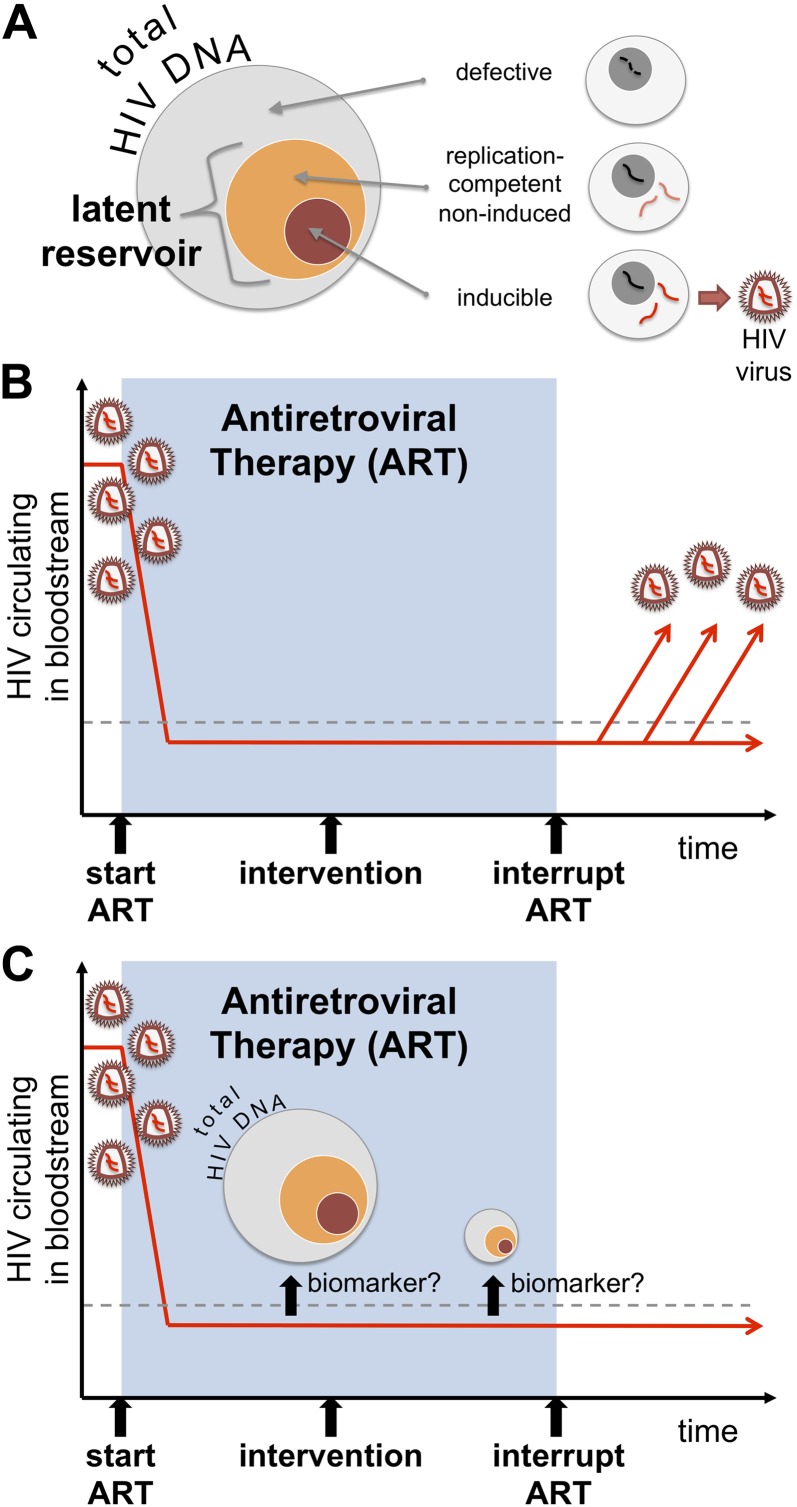
Identifying a biomarker for the latent reservoir of HIV could be used to guide future clinical trials. (**A**) Much of the HIV DNA harboured by infected cells is defective and cannot replicate itself, but there is a “latent reservoir” of intact HIV DNA that can support replication. However, it is difficult to measure the size of the latent reservoir because only a fraction of the cells in the reservoir can be readily induced to produce HIV in tissue cultures. (**B**) Antiretroviral therapy (or ART) can suppress the levels of HIV circulating in a patient's bloodstream (red line), below detectable levels (dashed line). However, the only way to currently determine whether a potential cure intervention (vertical black arrow) is effective or not is to interrupt ART and then monitor the amount of the virus in the blood to see if and when the virus ‘rebounds’. (**C**) Williams, Hurst et al. reveal that measuring the total level of HIV DNA can help predict the progress of an infection after ART is stopped. As such, total HIV DNA could be used as a predictive biomarker in future clinical trials testing potential cure interventions. The total DNA could be measured before a potential cure was administered, and then measured again some time afterwards to see if the level of total DNA has been decreased. Such a biomarker might allow clinicians to identify patients who can safely test cure interventions, and to select those who can safely interrupt their treatment if the ‘cure’ appears successful.

Now, in *eLife*, John Frater at the John Radcliffe Hospital in Oxford and co-workers in the SPARTAC collaboration report an important advance in the search for a biomarker for the latent reservoir. The SPARTAC study enrolled a cohort of individuals infected with HIV who were identified during the early stages of their infection. Subjects were randomly selected to receive either antiretroviral therapy for several weeks before the therapy was stopped, or no antiretroviral therapy (which is the standard course of action for an early HIV infection). This study aimed to determine if short-term ART during an early infection preserves the function of the immune system and eventually allows individuals to stop therapy safely. As previously reported (Fidler et al., 2013), the SPARTAC group found that this was not the case, and concluded that once ART is initiated it should not be interrupted.

A cure—or more accurately remission (a temporary end of disease symptoms)—will likely be defined based on how long a person remains free from replicating HIV in the absence of therapy. It is logical to predict that the smaller the HIV reservoir during ART, the longer it will take for the virus to rebound after the treatment is stopped (Hill et al., 2014): this idea is also supported by recent clinical experience (Yukl et al., 2013; Henrich et al., 2014; Persaud et al., 2013). A test that is inexpensive, sensitive and reproducible, and that accurately reflects the total amount of replication-competent HIV, would therefore have profound effects on cure research—particularly if it could predict what would happen if therapy were interrupted. Finding a biomarker that achieves all of this would allow investigators to perform efficient proof-of-concept studies and allow regulators to guide companies through the drug development process. Such a development would be comparable to the tremendous advances in antiviral therapies that were enabled by developing the HIV plasma viral load test, which measures how much virus is circulating in the bloodstream.

One way to estimate the size of the HIV latent reservoir would be to measure how many cells contain HIV DNA. In the SPARTAC study, Frater and co-workers—who include James Williams and Jacob Hurst as joint first authors—measured the frequency of certain white blood cells (the CD4^+^ T cells) that harbour HIV DNA (that is, the ‘total HIV DNA’) in all the participants at the start of the trial. Even though it is well recognised that most HIV DNA is defective and unable to support replication, the total DNA in early untreated HIV infection could predict how the disease would progress (Williams et al., 2014). The total DNA declined by several fold during the course of ART, as expected. The total DNA prior to stopping ART also predicted how quickly the number of CD4^+^ T cells would decline and how quickly the virus would rebound. Those patients with high levels of total DNA had over double the rate of viral rebound as individuals with low levels of total DNA. Total HIV DNA levels can thus predict how long a patient can safely remain off treatment, and may prove to be useful in monitoring potential cure treatments (Figure 1C). Studies to confirm these observations are now being planned.

Developing and evaluating possible HIV cures faces many challenges beyond how best to measure the reservoir. Nevertheless, there are now many interventions that might provide a cure, including early ART (Persaud et al., 2013; Saez-Cirion et al., 2013) and drugs that essentially shock the virus out of hiding (Archin et al., 2012). Other possible cures include gene therapies (Tebas et al., 2014), stem cell transplantation (Hutter et al., 2009; Henrich et al., 2014) and drugs that make the patients cells less hospitable for the virus (Barouch and Deeks, 2014). As such, progress towards a cure may not have stalled but it is certainly being hindered by the lack of a robust biomarker for the HIV reservoir. The findings by the SPARTAC team have now helped the field identify a way to move forward in finding such a biomarker.
